# Crystal structures of two coordination isomers of copper(II) 4-sulfo­benzoic acid hexa­hydrate and two mixed silver/potassium 4-sulfo­benzoic acid salts

**DOI:** 10.1107/S2056989019014610

**Published:** 2019-10-31

**Authors:** Philip J. Squattrito, Kelly J. Lambright-Mutthamsetty, Patrick A. Giolando, Kristin Kirschbaum

**Affiliations:** a Department of Chemistry and Biochemistry, Central Michigan University, Mount Pleasant, Michigan 48859, USA; b College of Natural Sciences and Mathematics, University of Toledo, Toledo, OH 43606, USA

**Keywords:** crystal structure, 4-sulfo­benzoic acid, mixed silver/potassium

## Abstract

A reaction of copper(II) carbonate and potassium 4-sulfo­benzoic acid in water acidified with hydro­chloric acid yielded two crystalline products. Tetra­aqua­bis­(4-carb­oxy­benzene­sulfonato)­copper(II) dihydrate, [Cu(O_3_SC_6_H_4_CO_2_H)_2_(H_2_O)_4_]·2H_2_O, (I), crystallizes in the triclinic space group *P*  with the Cu^2+^ ions located on centers of inversion. Each copper ion is coordinated to four water mol­ecules in a square plane with two sulfonate O atoms in the apical positions of a Jahn–Teller-distorted octa­hedron. The carboxyl­ate group is protonated and not involved in coordination to the metal ions. The complexes pack so as to create a layered structure with alternating inorganic and organic domains. The packing is reinforced by several O—H⋯O hydrogen bonds involving coordinated and non-coordinated water mol­ecules, the carb­oxy­lic acid group and the sulfonate group. Hexa­aqua­copper(II) 4-carb­oxy­benzene­sulfonate, [Cu(H_2_O)_6_](O_3_SC_6_H_4_CO_2_H)_2_, (II), also crystallizes in the triclinic space group *P*  with Jahn–Teller-distorted octa­hedral copper(II) aqua complexes on the centers of inversion. As in (I), the carboxyl­ate group on the anion is protonated and the structure consists of alternating layers of inorganic cations and organic anions linked by O—H⋯O hydrogen bonds. A reaction of silver nitrate and potassium 4-sulfo­benzoic acid in water also resulted in two distinct products that have been structurally characterized. An anhydrous silver potassium 4-carb­oxy­benzene­sulfonate salt, [Ag_0.69_K_0.31_](O_3_SC_6_H_4_CO_2_H), (III), crystallizes in the monoclinic space group *C*2/*c*. There are two independent metal sites, one fully occupied by silver ions and the other showing a 62% K^+^/38% Ag^+^ (fixed) ratio, refined in two slightly different positions. The coordination environments of the metal ions are composed primarily of sulfonate O atoms, with some participation by the non-protonated carboxyl­ate O atoms in the disordered site. As in the copper compounds, the cations and anions cleanly segregate into alternating layers. A hydrated mixed silver potassium 4-carb­oxy­benzene­sulfonate salt dihydrate, [Ag_0.20_K_0.80_](O_3_SC_6_H_4_CO_2_H)·2H_2_O, (IV), crystallizes in the monoclinic space group *P*2_1_/*c* with the Ag^+^ and K^+^ ions sharing one unique metal site coordinated by two water mol­ecules and six sulfonate O atoms. The packing in (IV) follows the dominant motif of alternating inorganic and organic layers. The protonated carboxyl­ate groups do not inter­act with the cations directly, but do participate in hydrogen bonds with the coordinated water mol­ecules. (IV) is isostructural with pure potassium 4-sulfo­benzoic acid dihydrate.

## Abstract 2

A reaction of copper(II) carbonate and potassium 4-sulfo­benzoic acid in water acidified with hydro­chloric acid yielded two crystalline products, tetra­aqua­bis­(4-carb­oxy­benzene­sulfonato)­copper(II) dihydrate and hexa­aqua­copper(II) 4-carb­oxy­benzene­sulfonate. A reaction of silver nitrate and potassium 4-sulfo­benzoic acid in water also resulted in two distinct products, an anhydrous silver potassium 4-carb­oxy­benzene­sulfonate salt and a hydrated mixed silver potassium 4-carb­oxy­benzene­sulfonate salt dihydrate.

## Chemical context   

Over the past few decades, organo­sulfonate and organo­carboxyl­ate anions have become popular building blocks for metal-organic framework (MOF) structures (Dey *et al.*, 2014 ▸; Shimizu *et al.*, 2009 ▸; Cai, 2004 ▸). Having previously investigated some structures of the bifunctional 4-sulfo­benzoic acid anion (Gunderman & Squattrito, 1994 ▸), we recently decided to examine its inter­actions with some softer (and therefore sulfophilic) late transition metals. Reactions with Cu^2+^ and Ag^+^ were carried out that resulted in four new structures that are described herein.
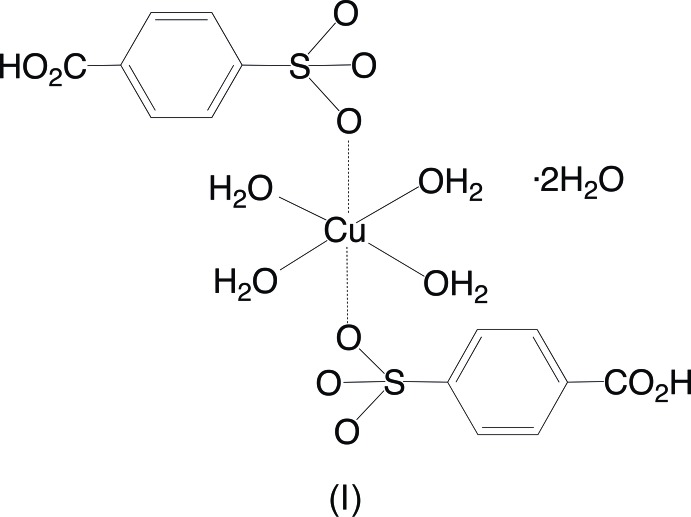
 
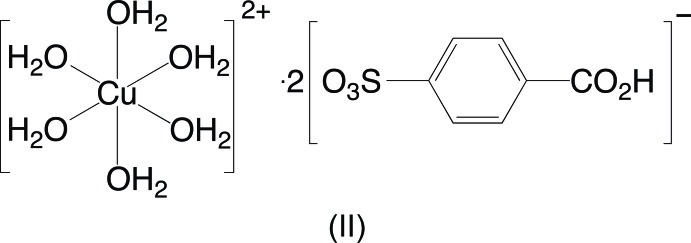
 
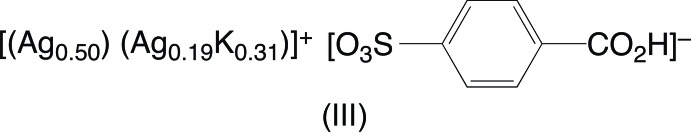
 
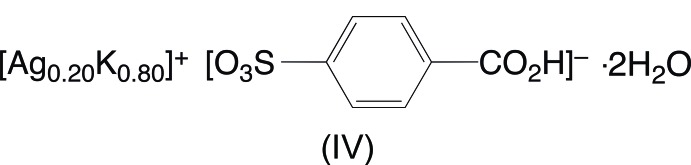


## Structural commentary   

**Figure 1:**
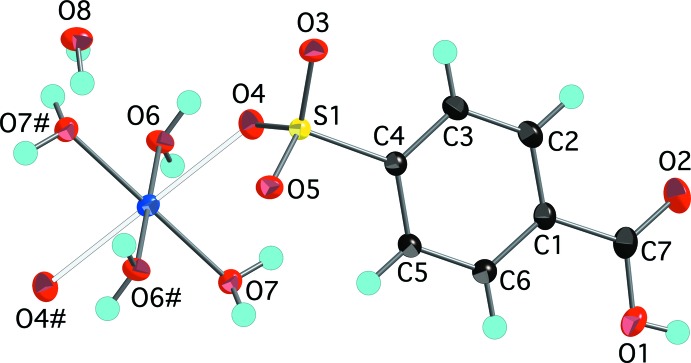
The mol­ecular structure of (I), showing the atom-numbering scheme. Displacement ellipsoids are shown at the 70% probability level and hydrogen atoms are shown as small spheres of arbitrary radii. Symmetry-equivalent water mol­ecules and the sulfonate O4 atom are included to show the complete coordination environment of the cation. The longer Jahn–Teller distorted Cu1—O4 distances are shown as hollow bonds. [Symmetry code: (#) 1 − *x*, 1 − *y*, −*z*.]

**Figure 2:**
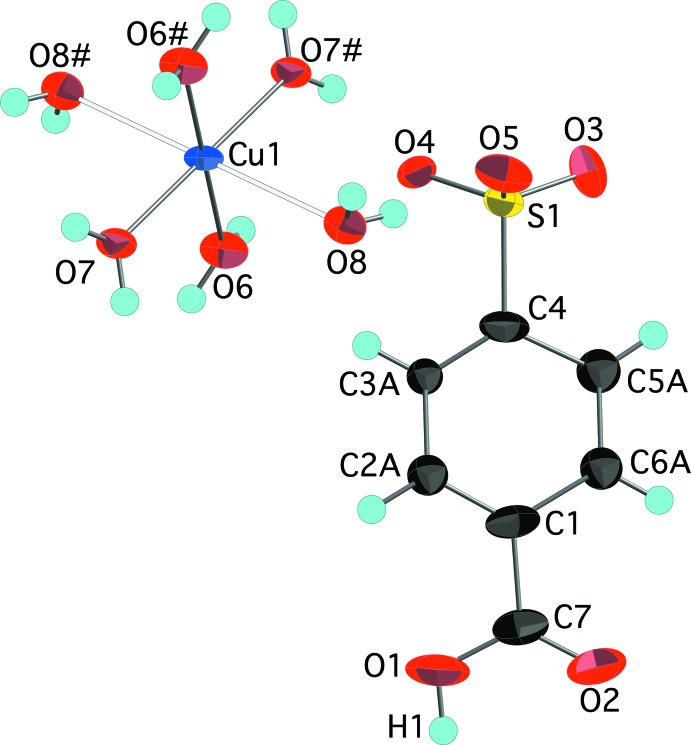
The mol­ecular structure of (II), showing the atom-numbering scheme. Displacement ellipsoids are shown at the 70% probability level and hydrogen atoms are shown as small spheres of arbitrary radii. Only one of the disordered orientations of the arene ring (atoms C2*A*—C6*A* at 50% occupancy) is shown. Symmetry-equivalent water mol­ecules are included to show the complete coordination environment of the cation. The longer Jahn–Teller-distorted Cu1—O8 distances are shown as hollow bonds. [Symmetry code: (#) 1 − *x*, 2 − *y*, −*z*.]

The aqueous reaction of copper(II) carbonate, potassium 4-sulfo­benzoic acid, and hydro­chloric acid produced two copper-containing products. Blue parallelepiped-shaped crystals were found to have the formula [Cu(H_2_O)_4_(O_3_SC_6_H_4_CO_2_H)_2_]·2H_2_O, (I). The structure finds the Cu^2+^ ions on centers of inversion with four closely bound water mol­ecules [Cu—O distances of 1.9520 (7) and 1.9743 (7) Å] in a square plane [O6—Cu1—O7 angle of 90.38 (3)°] (Fig. 1 ▸). Two sulfonate O atoms at 2.3934 (8) Å occupy the apical positions to complete a classic Jahn–Teller-distorted octa­hedral coordination of the copper ion. This type of bis­(sulfanato)copper(II) complex with the sulfonate ligands in the more distant apical position has been reported by Cai *et al.* (2001 ▸) with Cu—O distances *ca* 0.1–0.4 Å longer than the Cu1—O4 distance in (I). A comparable Cu—O sulfonate distance of 2.420 (2) Å is seen in bis­(4-amino­benzene­sulfonato)­diaqua­copper(II) (Gunderman *et al.*, 1996 ▸). The second product of the reaction, blue needles, was determined to be [Cu(H_2_O)_6_](O_3_SC_6_H_4_CO_2_H)_2_, (II), a structural isomer of (I). The copper ions in (II) are also centrosymmetric and Jahn–Teller distorted with four close [Cu—O distances of 1.941 (3) and 1.953 (3) Å] and two more distant [Cu—O = 2.515 (3) Å] water mol­ecules in an otherwise very regular octa­hedral geometry (Fig. 2 ▸). As in (I), the carboxyl­ate group is protonated and does not have any direct metal–oxygen inter­actions. The lack of metal–sulfonate bonding is more typical of the behavior of other 3*d*-block divalent transition metals (Leonard *et al.*, 1999 ▸).

**Figure 3:**
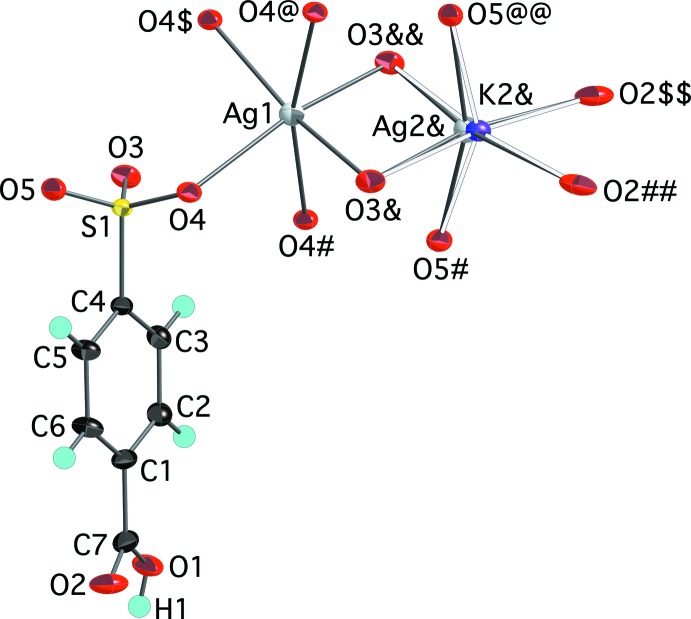
The mol­ecular structure of (III), showing the atom-numbering scheme. Displacement ellipsoids are shown at the 70% probability level and hydrogen atoms are shown as small spheres of arbitrary radii. Symmetry-equivalent oxygen atoms are included to show the complete coordination environments of the cations. Atoms Ag2 and K2 are present at 38% and 62% occupancies. The K2—O inter­actions are shown as hollow bonds for clarity. [Symmetry codes: ($) 1 − *x*, *y*, 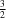 − *z*; (&&) 1 − *x*, 1 − *y*, 1 − *z*; (@) 1 − *x*, 1 − *y*, 2 − *z*; (#) *x*, 1 − *y*, *z* − 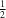; (&) *x*, 1 − *y*, *z* + 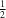; (@@) 1 − *x*, 1 − *y*, 2 − *z*; ($$) *x* + 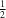, *y* + 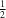, *z*; (##) 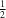 − *x*, *y* + 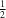, 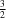 − *z*.]

The reaction of silver nitrate and potassium 4-sulfo­benzoic acid yielded two silver-containing crystalline products reported here. Colorless needle-shaped crystals were identified as Ag_0.69_K_0.31_(O_3_SC_6_H_4_CO_2_H), (III), an anhydrous mixed silver/potassium salt of 4-sulfo­benzoic acid. The asymmetric unit (Fig. 3 ▸) contains two independent cation sites, both on twofold symmetry special positions of the space group *C*2/*c*. One site (Ag1) was judged to be fully occupied by Ag^+^ cations, while the other site consists of split positions *ca* 0.2 Å apart. This site was modeled as two positions (Ag2 and K2) with partial occupancies fixed at 38% and 62%, respectively. The overall composition of the data crystal is 69% Ag and 31% K, which was corroborated by energy dispersive X-ray (EDX) analysis. Ag1 is coordinated by six sulfonate O atoms at distances ranging from 2.4919 (11) to 2.5061 (10) Å in a moderately distorted octa­hedral geometry. Ag2 and K2 are also in a distorted octa­hedral environment formed from four sulfonate and two carboxyl­ate O atoms at distances of 2.470 (3)–2.751 (3) Å (Ag2) and 2.584 (6)–2.653 (2) Å (K2). The Ag—O distances are consistent with those seen in other silver arene­sulfonates (Côté & Shimizu, 2004 ▸), while the K—O distances are slightly shorter than those seen in three polymorphs of potassium 4-sulfo­benzoic acid (Kariuki & Jones, 1995 ▸), which are mostly between *ca* 2.65 and 2.95 Å. The extensive metal–sulfonate bonding is as expected given the softer nature of Ag^+^ and K^+^ relative to divalent 3*d* transition metal ions (Parr & Pearson, 1983 ▸). As in (I) and (II), the carboxyl­ate group remains protonated with the acidic H atom unambiguously located on O1.

**Figure 4:**
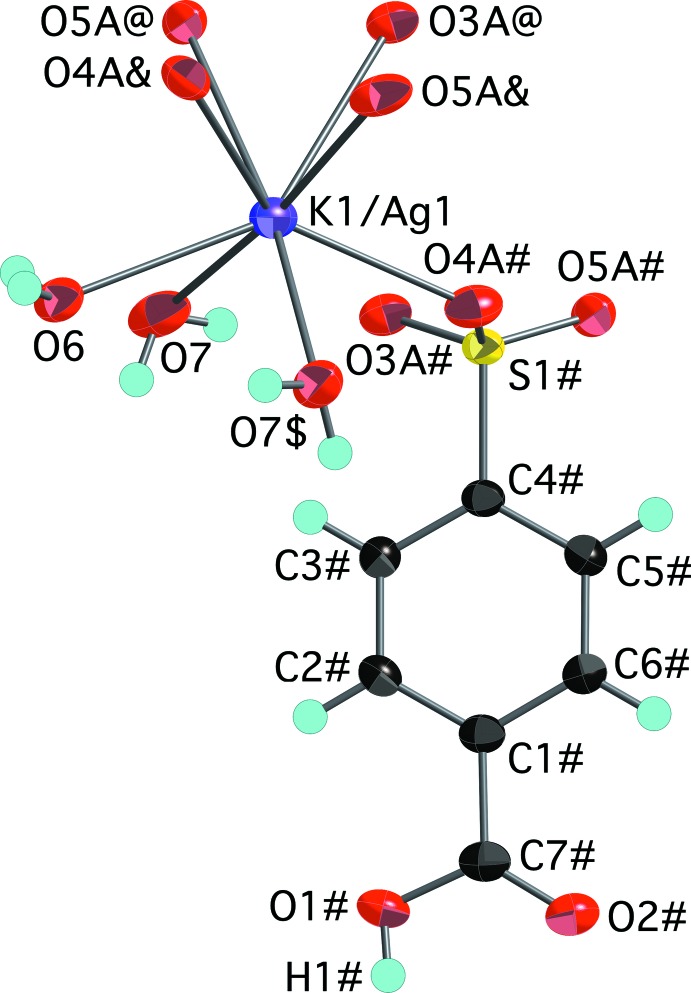
The mol­ecular structure of (IV), showing the atom-numbering scheme. Displacement ellipsoids are shown at the 70% probability level and hydrogen atoms are shown as small spheres of arbitrary radii. Symmetry-equivalent water mol­ecules and sulfonate oxygen atoms are included to show the complete coordination environment of the cation. The minor disordered component of the sulfonate group (atoms O3*B*, O4*B*, and O5*B*) has been omitted for clarity. [Symmetry codes: ($) *x*, 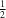 − *y*, *z* − 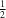; (&) 1 + *x*, *y*, *z*; (@) 1 + *x*, −*y* + 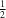, *z* + 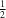; (#) 1 − *x*, *y* − 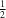, 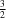 − *z*.]

The second product of the silver reaction crystallizes as colorless hexa­gonal plates determined to be Ag_0.20_K_0.80_(O_3_SC_6_H_4_CO_2_H)·2H_2_O, (IV). This compound is isostructural with K(O_3_SC_6_H_4_CO_2_H)·2H_2_O, one of the polymorphs of the starting material potassium 4-sulfo­benzoic acid whose structure has been reported (Gunderman & Squattrito, 1994 ▸; Kariuki & Jones, 1995 ▸). The unique cation site was modeled as disordered with Ag^+^ and K^+^ present at occupancies fixed at 20% and 80%, respectively. This composition is supported by EDX analysis of the data crystal. The cation is surrounded by eight O atoms, including three water mol­ecules and five sulfonate O atoms (Fig. 4 ▸). Although Shannon (1976 ▸) assigns Ag^+^ a smaller radius than K^+^, they are within 15–20% of each other for coordination number 8 so occupancy of the same site seems reasonable. The K1/Ag1-O_water_ distances [2.6233 (12), 2.7045 (13) and 2.8017 (11) Å] are *ca* 0.09 Å shorter than those reported for the site fully occupied by K^+^, however, both determinations of the latter used room temperature data so the difference cannot be directly attributed to the smaller radius of the Ag^+^ ion. The tendency of potassium and silver to occupy the same or similar sites in the arene sulfonate/carboxyl­ate structures observed in this study is not the rule. For example, in silver potassium 5-sulfosalicylic acid, the Ag^+^and K^+^ ions occupy separate sites in the structure with very different coordination environments and no indication of mixed-occupancy (Li *et al.*, 2006 ▸).

## Supra­molecular features   

**Figure 5:**
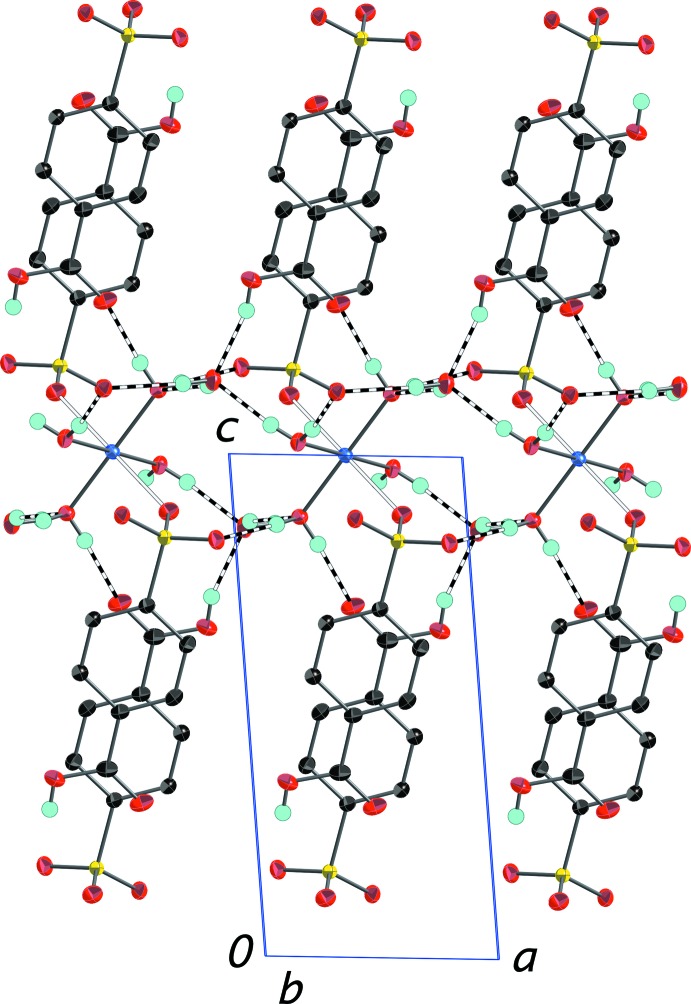
Packing diagram of (I) with the outline of the unit cell. View is onto the (010) plane. O—H⋯O hydrogen bonds connecting the layers of copper complexes are shown as dashed bonds. H atoms bonded to C atoms have been omitted. The longer Jahn–Teller-distorted Cu1—O4 distances are shown as hollow bonds. Displacement ellipsoids are drawn at the 70% probability level.

**Figure 6:**
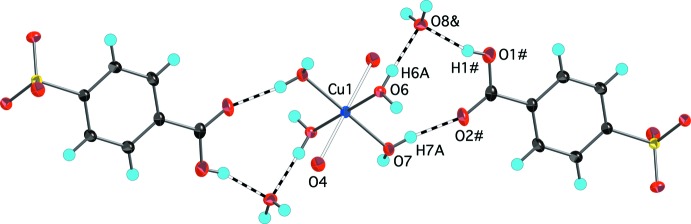
Partial packing diagram of (I) showing a portion of the hydrogen-bonding scheme involving coordinated water mol­ecules O6 and O7, non-coordinated water mol­ecule O8, and the carb­oxy­lic acid group. Hydrogen bonds are shown as dashed bonds. The longer Jahn–Teller-distorted Cu1—O4 distances are shown as hollow bonds. Displacement ellipsoids are drawn at the 70% probability level. [Symmetry codes: (#) 1 − *x*, 1 − *y*, 1 − *z*; (&) *x* − 1, *y*, *z*.]

The complexes in (I) pack so as to create distinct layers of copper ions in the *ab* plane that alternate with layers of 4-sulfo­benzoic acid anions stacking in the *c*-axis direction (Fig. 5 ▸). This two-dimensional alternating inorganic–organic motif is typical of metal arene­sulfonates reported by us (Gunderman *et al.*, 1996 ▸; Leonard *et al.*, 1999 ▸) and others (Cai, 2004 ▸). The carboxyl­ate group remains protonated with the H atom clearly located on atom O1 and the CO_2_H moieties are situated within the organic layer with no direct inter­action with the cations. An extensive network of strong, nearly linear O—H⋯O hydrogen bonds (Table 1 ▸) involving the carb­oxy­lic H atom, coordinated water mol­ecules, unprotonated sulfonate and carboxyl­ate O atoms, and a non-coordinated water mol­ecule reinforce the packing. A portion of this network is shown in more detail in Fig. 6 ▸.

**Figure 7:**
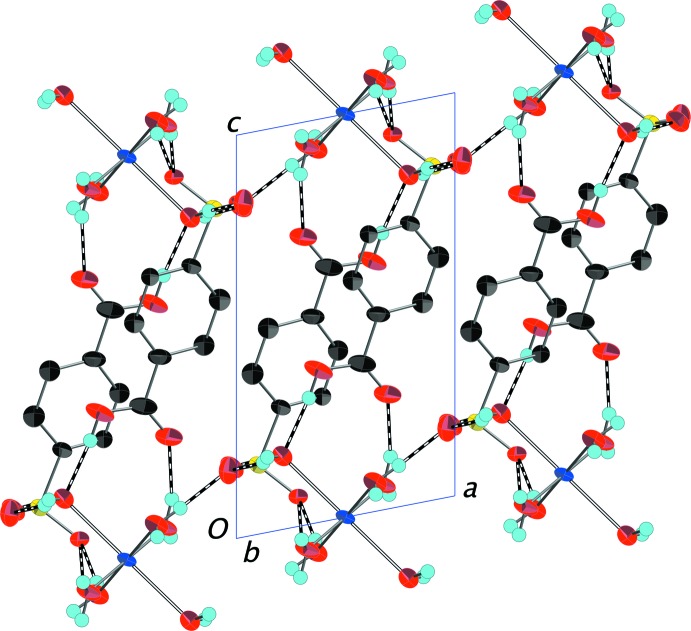
Packing diagram of (II) with the outline of the unit cell. View is onto the (010) plane. Only one of the disordered orientations of the arene rings (at 50% occupancy) is shown. O—H⋯O hydrogen bonds connecting the layers of hexa­aqua­copper complexes and 4-sulfo­benzoic acid anions are shown as dashed bonds. H atoms bonded to C atoms have been omitted. The longer Jahn–Teller-distorted Cu1—O8 distances are shown as hollow bonds. Displacement ellipsoids are drawn at the 70% probability level.

The packing pattern in (II) is very similar to that in (I) with layers of hexa­aqua­copper(II) cations in the *ab* plane alternating with layers of 4-sulfo­benzoic acid anions along the *c*-axis direction (Fig. 7 ▸). The anions are positioned with the sulfonate groups on the exterior of the layer and the carb­oxy­lic acid groups somewhat more to the inter­ior. All of the oxygen-bound H atoms participate in strong approximately linear O—H⋯O hydrogen bonds to the unprotonated sulfonate and carboxyl­ate O atoms or in the case of the carb­oxy­lic H atom to a coordinated water O atom (Table 2 ▸).

Given the highly acidic conditions of the reaction, it is not surprising that the less acidic carboxyl­ate proton is present in both products, effectively preventing the carboxyl­ate group from bonding directly to the copper ions. This outcome is undesirable from the standpoint of using the difunctional anion as a building block to make more extended metal–organic frameworks. Studies by other workers have shown that the use of hydro­thermal conditions at higher pH can be an effective route to novel structures of aromatic sulfonate/carboxyl­ate anions with coordination by both groups (Sun *et al.*, 2004 ▸). Other studies have successfully produced the desired framework structures without the need for hydro­thermal methods (Kurc *et al.*, 2012 ▸).

**Figure 8:**
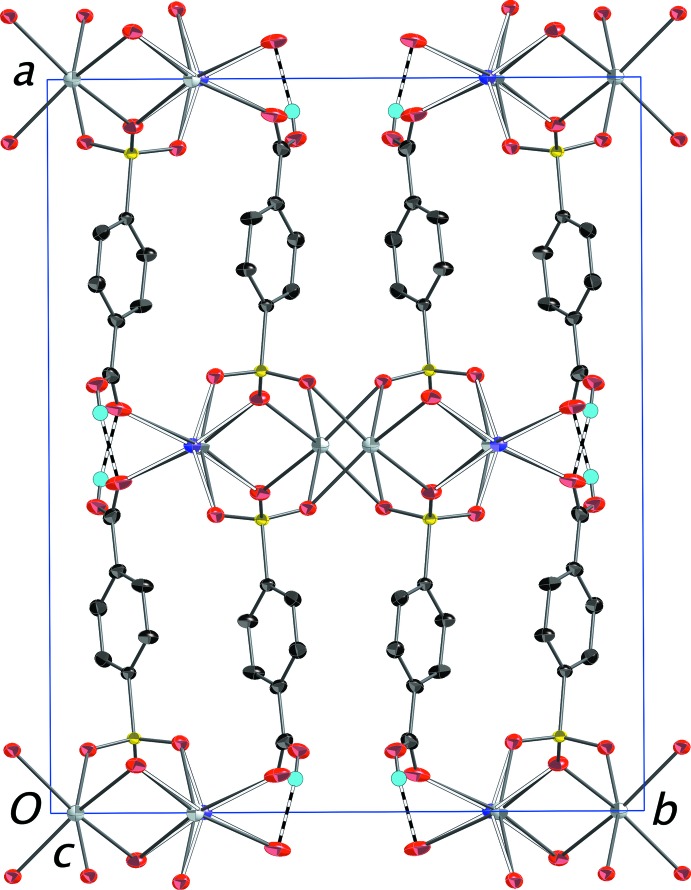
Packing diagram of (III) with the outline of the unit cell. View is onto the (001) plane. The layers of 4-sulfo­benzoic acid anions are evident with the silver and potassium ions situated in between the layers. O—H⋯O hydrogen bonds connecting the carb­oxy­lic H atoms and carboxyl­ate O atoms of adjacent layers are shown as dashed bonds. H atoms bonded to C atoms have been omitted. Displacement ellipsoids are drawn at the 70% probability level.

The packing in (III) features layers of metal ions in the *bc* plane alternating with layers of 4-sulfo­benzoic acid anions stacking along the *a*-axis direction (Fig. 8 ▸). Anions in adjacent layers are linked in part by O—H⋯O hydrogen bonds between neighboring carb­oxy­lic acid groups in the classic dimerization of such mol­ecules (Table 3 ▸). Since both functional groups are involved in metal bonding, the anions are positioned with both groups equally exterior with respect to the layer, in contrast to the slipped arrangement in (I) and (II).

**Figure 9:**
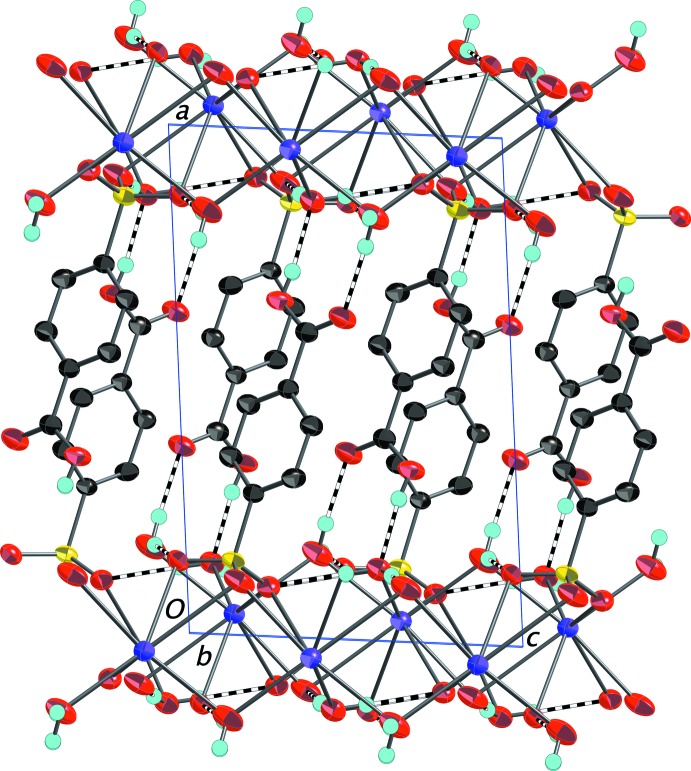
Packing diagram of (IV) with the outline of the unit cell. View is onto the (010) plane. The layers of 4-sulfo­benzoic acid anions are in the center of the cell with the silver and potassium ions (disordered over the same site) situated in between the layers. O—H⋯O hydrogen bonds between the carb­oxy­lic groups and coordinated water mol­ecules are shown as dashed bonds. H atoms bonded to C atoms have been omitted. Displacement ellipsoids are drawn at the 70% probability level.

As in the other structures reported here, the carb­oxy­lic acid in (IV) is protonated and as in (I) and (II), it is in a more inter­ior position in the anion layer than is the sulfonate group (Fig. 9 ▸). Once again, all of the oxygen-bound H atoms participate in a robust O—H⋯O network of hydrogen bonds detailed in Table 4 ▸.

## Synthesis and crystallization   

The reaction that produced (I) and (II) was commenced by dissolving 2.085 g (8.68 mmol) of potassium 4-sulfo­benzoic acid (Aldrich, 98%) in 60 ml of water with gentle heating and stirring. To this solution was added 1.053 g (8.52 mmol) CuCO_3_ (Fisher), creating a thick green opaque mixture, followed by 50 drops of 12 *M* HCl. The solid gradually dissolved over *ca* 3 h leaving a clear light-blue solution that was then transferred to a porcelain evaporating dish and set out in a fume hood. Five days later, the water had completely evaporated, leaving behind large qu­anti­ties of three types of crystals: large colorless to slightly yellow plates, light-blue needles, and small blue parallelepipeds. The colorless plates were identified to be potassium 4-sulfo­benzoic acid dihydrate, the structure of which has been reported (Gunderman & Squattrito, 1994 ▸; Kariuki & Jones, 1995 ▸). The blue parallelepipeds are (I) and the blue needles are (II).

A 2.012 g (8.37 mmol) sample of potassium 4-sulfo­benzoic acid (Aldrich, 98%) was dissolved in 50 ml of water with gentle heat and stirring. To this colorless solution was added a colorless solution of 1.420 g (8.36 mmol) of AgNO_3_ (Baker) in 25 ml of water. The resulting slightly turbid opalescent mixture was transferred to a porcelain evaporating dish that was set out to evaporate in a fume hood. During the transfer, some white snowy particles were noted in the liquid. After several days, the water had completely evaporated leaving behind colorless crystals of two distinct morphologies, needles and hexa­gonal plates. The needles were identified as (III) and the plates as (IV) through the single crystal X-ray studies.

## Refinement   

Crystal data, data collection and structure refinement details are summarized in Table 5 ▸. For (I), hydrogen atoms bonded to carbon atoms and the carb­oxy­lic hydrogen atom were calculated on idealized positions and included in the refinement as riding atoms with C—H = 0.95 Å or O—H = 0.78 Å and their *U*_iso_ constrained to be 1.2 (C—H) or 1.5 (O—H) times the *U*_eq_ of the bonding atom. Hydrogen atoms bonded to water oxygen atoms were located in difference-Fourier maps and refined, followed by restraining the O—H distance to be 0.84 Å (DFIX) and constraining their *U*_iso_ to be 1.5 times the *U*_eq_ of the bonding atom. All crystals of (II) under investigation exhibited twinning and the structure was refined as a two-component twin with a 0.523 (2):0.477 (2) ratio. The twinning law was determined to be a 180° rotation around the triclinic *b* axis. Additionally, the arene rings are statistically disordered over two orientations such that atoms C2, C3, C5, and C6 are split between two positions (designated *A* and *B*) each assigned 50% occupancy. These atoms were refined with isotropic displacement parameters. All other non-hydrogen atoms were refined with anisotropic displacement parameters and full occupancies. The C—H hydrogen atoms were included as riding atoms with fixed distances of 0.93 Å. The O—H hydrogen atoms were located using difference-Fourier syntheses and were refined with their displacement parameters constrained to those of the bonding atoms (distances in Table 2 ▸). In (III), one of the two cation sites showed split positions separated by *ca* 0.2 Å. These were modeled as one containing Ag fixed at 38% occupancy and the other containing K fixed at 62% occupancy. With the other cation site modeled as 100% Ag, the overall composition of the data crystal based on the refinement is Ag_0.69_K_0.31_(O_3_SC_6_H_4_CO_2_H). Energy dispersive X-ray analysis (EDX) of three locations on the data crystal yielded an average Ag/K atom ratio matching the refinement composition. Hydrogen atoms bonded to carbon atoms were calculated on idealized positions and included in the refinement as riding atoms (C—H 0.95Å) with their *U*_iso_ constrained to be 1.2 times the *U*_eq_ of the bonding atom. The carb­oxy­lic hydrogen atom was placed on an idealized position with consideration given to the maximum of the electron density. It was then refined as a rotating group (around C7—O1) and *U*_iso_ was fixed to 1.5 times the *U*_eq_ of the bonding atom O1. In (IV), the unique cation site was modeled with a fixed 80% K/20% Ag occupancy constraining fractional coordinates and atomic displacement parameters to be the same for Ag and K. Energy dispersive X-ray analysis (EDX) of three locations on the data crystal yielded an average K/Ag atom ratio in reasonable agreement with this 4:1 ratio. In addition, the sulfonate group displayed disorder with two sets of O atom positions (designated *A* and *B*) separated by an approximate 12° rotation about the C—S bond. The occupancies were assigned as 80% *A* and 20% *B*, with the *A* atoms being refined anisotropically and the *B* atoms isotropically. All other non-hydrogen atoms were refined anisotropically. Hydrogen atoms bonded to carbon atoms were calculated on idealized positions and included in the refinement as riding atoms (C—H = 0.95Å) with their *U*_iso_ constrained to be 1.2 times the *U*_eq_ of the bonding atom. The carboxyl hydrogen atom was placed on an idealized position with consideration given to the maximum of the electron density. It was then refined as a rotating group (around C7—O1) and *U*_iso_ was fixed to 1.5 times the *U*_eq_ of the bonding atom O1. Water hydrogen atoms were located in difference-Fourier maps and refined, followed by restraining the O—H distance to be 0.84 Å (DFIX) and constraining their *U*_iso_ to be 1.5 times the *U*_eq_ of the bonding atom.

## Supplementary Material

Crystal structure: contains datablock(s) I, II, III, IV, global. DOI: 10.1107/S2056989019014610/mw2148sup1.cif

Structure factors: contains datablock(s) I. DOI: 10.1107/S2056989019014610/mw2148Isup2.hkl

Structure factors: contains datablock(s) II. DOI: 10.1107/S2056989019014610/mw2148IIsup3.hkl

Structure factors: contains datablock(s) III. DOI: 10.1107/S2056989019014610/mw2148IIIsup4.hkl

Structure factors: contains datablock(s) IV. DOI: 10.1107/S2056989019014610/mw2148IVsup5.hkl

CCDC references: 1961811, 1961810, 1961809, 1961808, 1961811, 1961810, 1961809, 1961808

Additional supporting information:  crystallographic information; 3D view; checkCIF report

## Figures and Tables

**Table 1:** Hydrogen-bond geometry (Å, °) for (I)

*D*—H⋯*A*	*D*—H	H⋯*A*	*D*⋯*A*	*D*—H⋯*A*
O1—H1⋯O8^i^	0.78	1.94	2.6979 (12)	164
O6—H6*B*⋯O5^ii^	0.81 (1)	1.97 (1)	2.7738 (11)	172 (2)
O6—H6*A*⋯O8^iii^	0.81 (1)	1.88 (1)	2.6872 (11)	172 (2)
O7—H7*A*⋯O2^iv^	0.83 (1)	1.86 (1)	2.6845 (11)	171 (2)
O7—H7*B*⋯O3^v^	0.84 (1)	1.84 (1)	2.6672 (11)	169 (2)
O8—H8*B*⋯O7	0.82 (1)	2.17 (1)	2.9255 (11)	153 (2)
O8—H8*A*⋯O5^ii^	0.83 (1)	1.99 (1)	2.7984 (11)	165 (2)

Symmetry codes: (i) 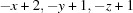; (ii) 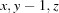; (iii) 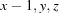; (iv) 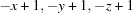; (v) 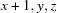.

**Table 2:** Hydrogen-bond geometry (Å, °) for (II)

*D*—H⋯*A*	*D*—H	H⋯*A*	*D*⋯*A*	*D*—H⋯*A*
O1—H1⋯O8	0.86 (7)	1.83 (7)	2.677 (4)	170 (7)
O6—H61⋯O5^i^	0.84 (2)	1.89 (3)	2.717 (4)	169 (5)
O6—H62⋯O4^ii^	0.84 (2)	1.93 (3)	2.725 (5)	158 (5)
O7—H71⋯O4^iii^	0.83 (2)	1.99 (3)	2.784 (5)	160 (5)
O7—H72⋯O2	0.83 (2)	1.84 (3)	2.645 (4)	161 (5)
O8—H81⋯O3^iv^	0.85 (2)	2.02 (3)	2.851 (5)	167 (5)
O8—H82⋯O5^v^	0.84 (2)	2.02 (3)	2.854 (5)	175 (5)

Symmetry codes: (i) 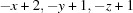; (ii) 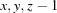; (iii) 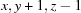; (iv) 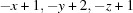; (v) 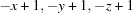.

**Table 3:** Hydrogen-bond geometry (Å, °) for (III)

*D*—H⋯*A*	*D*—H	H⋯*A*	*D*⋯*A*	*D*—H⋯*A*
O1—H1⋯O2^i^	0.75	1.94	2.6841 (16)	172

Symmetry code: (i) 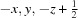.

**Table 4:** Hydrogen-bond geometry (Å, °) for (IV)

*D*—H⋯*A*	*D*—H	H⋯*A*	*D*⋯*A*	*D*—H⋯*A*
O1—H1⋯O6	0.79	1.85	2.6328 (14)	168
O6—H6*A*⋯O5*A* ^i^	0.85 (1)	2.01 (1)	2.840 (3)	168 (2)
O6—H6*A*⋯O5*B* ^i^	0.85 (1)	2.01 (2)	2.819 (12)	159 (2)
O6—H6*B*⋯O4*A* ^ii^	0.84 (1)	1.99 (1)	2.824 (3)	176 (2)
O6—H6*B*⋯O4*B* ^ii^	0.84 (1)	1.82 (2)	2.643 (12)	168 (2)
O7—H7*A*⋯O2	0.84 (1)	1.97 (1)	2.8111 (15)	177 (2)
O7—H7*B*⋯O3*A* ^iii^	0.84 (1)	2.03 (1)	2.838 (3)	162 (2)
O7—H7*B*⋯O3*B* ^iii^	0.84 (1)	1.87 (2)	2.650 (12)	155 (2)

Symmetry codes: (i) 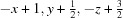; (ii) 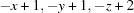; (iii) 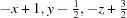.

**Table 5:** Experimental details

	(I)	(II)	(III)	(IV)
Crystal data				
Chemical formula	[Cu(C_7_H_5_O_5_S)_2_(H_2_O)_4_]·2H_2_O	[Cu(H_2_O)_6_](C_7_H_5_O_5_S)_2_	[Ag_0.69_K_0.31_](C_7_H_5_O_5_S)	[Ag_0.20_K_0.80_](C_7_H_5_O_5_S)·2H_2_O
*M*_r_	573.97	573.97	287.72	290.06
Crystal system, space group	Triclinic, *P* 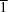	Triclinic, *P* 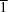	Monoclinic, *C*2/*c*	Monoclinic, *P*2_1_/*c*
Temperature (K)	130	130	120	120
*a*, *b*, *c* (Å)	6.1907 (1), 7.2010 (2), 12.4919 (3)	6.4380 (13), 7.2431 (14), 12.088 (2)	19.436 (3), 15.644 (3), 5.3355 (9)	12.8018 (7), 9.9170 (6), 8.4013 (5)
α, β, γ (°)	90.310 (1), 94.587 (1), 111.087 (1)	72.60 (3), 77.20 (3), 82.13 (3)	90, 95.651 (2), 90	90, 94.747 (1), 90
*V* (Å^3^)	517.57 (2)	523.0 (2)	1614.4 (5)	1062.93 (11)
*Z*	1	1	8	4
Radiation type	Mo *K*α	Mo *K*α	Mo *K*α	Mo *K*α
μ (mm^−1^)	1.34	1.33	2.18	0.99
Crystal size (mm)	0.14 × 0.12 × 0.06	0.21 × 0.08 × 0.02	0.16 × 0.06 × 0.03	0.23 × 0.17 × 0.07

Data collection				
Diffractometer	Bruker APEXII CCD	Bruker APEXII CCD	Bruker APEXII CCD	Bruker APEXII CCD
Absorption correction	Multi-scan (*SADABS*; Krause *et al.*, 2015 ▸)	Multi-scan (*TWINABS*; Sheldrick, 1996 ▸)	Multi-scan (*SADABS*; Krause *et al.*, 2015 ▸)	Multi-scan (*SADABS*; Krause *et al.*, 2015 ▸)
*T*_min_, *T*_max_	0.685, 0.747	0.585, 0.747	0.572, 0.648	0.666, 0.746
No. of measured, independent and observed [*I* > 2σ(*I*)] reflections	13360, 3630, 3460	3738, 3738, 3370	12751, 2435, 2223	16575, 3255, 2831
*R*_int_	0.011	0.045	0.020	0.023
(sin θ/λ)_max_ (Å^−1^)	0.767	0.766	0.712	0.716

Refinement				
*R*[*F* ^2^ > 2σ(*F* ^2^)], *wR*(*F* ^2^), *S*	0.020, 0.057, 1.14	0.059, 0.139, 1.18	0.017, 0.043, 1.08	0.025, 0.063, 1.08
No. of reflections	3630	3738	2435	3255
No. of parameters	171	169	135	171
No. of restraints	6	18	0	4
H-atom treatment	H atoms treated by a mixture of independent and constrained refinement	H atoms treated by a mixture of independent and constrained refinement	H atoms treated by a mixture of independent and constrained refinement	H atoms treated by a mixture of independent and constrained refinement
Δρ_max_, Δρ_min_ (e Å^−3^)	0.51, −0.43	0.84, −1.19	0.51, −0.36	0.44, −0.54

Computer programs: *APEX3* and *SAINT* (Bruker, 2015 ▸), *SHELXT2018* (Sheldrick, 2015*a* ▸), *SHELXL2017* (Sheldrick, 2015*b* ▸), *CrystalMaker* (Palmer, 2014 ▸) and *CELL_NOW 2008/4* (Sheldrick, 2008 ▸).

## supplementary crystallographic information

### Tetraaquabis(4-carboxybenzenesulfonato)copper(II) dihydrate (I) Crystal data

**Table:**

[Cu(C_7_H_5_O_5_S)_2_(H_2_O)_4_]·2H_2_O	*Z* = 1
*M**_r_* = 573.97	*F*(000) = 295
Triclinic, *P*1	*D*_x_ = 1.841 Mg m^−^^3^
*a* = 6.1907 (1) Å	Mo *K*α radiation, λ = 0.71073 Å
*b* = 7.2010 (2) Å	Cell parameters from 9988 reflections
*c* = 12.4919 (3) Å	θ = 3.0–32.9°
α = 90.310 (1)°	µ = 1.34 mm^−^^1^
β = 94.587 (1)°	*T* = 130 K
γ = 111.087 (1)°	Parallelpiped, light blue
*V* = 517.57 (2) Å^3^	0.14 × 0.12 × 0.06 mm

### Tetraaquabis(4-carboxybenzenesulfonato)copper(II) dihydrate (I) Data collection

**Table:**

Bruker APEXII CCD diffractometer	3460 reflections with *I* > 2σ(*I*)
Radiation source: sealed tube	*R*_int_ = 0.011
φ and ω scans	θ_max_ = 33.0°, θ_min_ = 1.6°
Absorption correction: multi-scan (SADABS; Krause *et al.*, 2015)	*h* = −9→9
*T*_min_ = 0.685, *T*_max_ = 0.747	*k* = −10→10
13360 measured reflections	*l* = −18→18
3630 independent reflections	

### Tetraaquabis(4-carboxybenzenesulfonato)copper(II) dihydrate (I) Refinement

**Table:**

Refinement on *F*^2^	Primary atom site location: dual
Least-squares matrix: full	Secondary atom site location: difference Fourier map
*R*[*F*^2^ > 2σ(*F*^2^)] = 0.020	Hydrogen site location: mixed
*wR*(*F*^2^) = 0.057	H atoms treated by a mixture of independent and constrained refinement
*S* = 1.14	*w* = 1/[σ^2^(*F*_o_^2^) + (0.0245*P*)^2^ + 0.273*P*] where *P* = (*F*_o_^2^ + 2*F*_c_^2^)/3
3630 reflections	(Δ/σ)_max_ = 0.001
171 parameters	Δρ_max_ = 0.51 e Å^−^^3^
6 restraints	Δρ_min_ = −0.43 e Å^−^^3^

### Tetraaquabis(4-carboxybenzenesulfonato)copper(II) dihydrate (I) Special details

**Table:**

Geometry. All esds (except the esd in the dihedral angle between two l.s. planes) are estimated using the full covariance matrix. The cell esds are taken into account individually in the estimation of esds in distances, angles and torsion angles; correlations between esds in cell parameters are only used when they are defined by crystal symmetry. An approximate (isotropic) treatment of cell esds is used for estimating esds involving l.s. planes.

### Tetraaquabis(4-carboxybenzenesulfonato)copper(II) dihydrate (I) Fractional atomic coordinates and isotropic or equivalent isotropic displacement parameters (Å^2^)

**Table:**

	*x*	*y*	*z*	*U*_iso_*/*U*_eq_	
Cu1	0.500000	0.500000	0.000000	0.00968 (5)	
S1	0.30355 (4)	0.79731 (3)	0.16948 (2)	0.00914 (5)	
O5	0.48130 (13)	0.96708 (11)	0.12629 (6)	0.01327 (14)	
O4	0.27720 (14)	0.60777 (11)	0.11718 (6)	0.01404 (14)	
O3	0.08255 (13)	0.82667 (12)	0.17228 (6)	0.01368 (14)	
O2	0.48509 (16)	0.66679 (14)	0.69906 (7)	0.02187 (17)	
O1	0.85182 (15)	0.79258 (14)	0.65304 (7)	0.01982 (17)	
H1	0.8822 (8)	0.785 (3)	0.7143 (16)	0.030*	
O6	0.29941 (13)	0.22730 (11)	0.02506 (6)	0.01153 (13)	
H6B	0.363 (3)	0.154 (2)	0.0511 (13)	0.017*	
H6A	0.187 (2)	0.212 (2)	0.0583 (12)	0.017*	
O7	0.71588 (13)	0.49375 (12)	0.12393 (6)	0.01239 (13)	
H7A	0.646 (3)	0.452 (2)	0.1780 (10)	0.019*	
H7B	0.818 (2)	0.6062 (16)	0.1396 (13)	0.019*	
O8	0.95280 (13)	0.20843 (12)	0.14612 (7)	0.01442 (14)	
H8B	0.930 (3)	0.3126 (18)	0.1349 (14)	0.022*	
H8A	0.8216 (19)	0.119 (2)	0.1378 (14)	0.022*	
C4	0.40161 (17)	0.78343 (14)	0.30569 (8)	0.01038 (16)	
C3	0.23888 (18)	0.71925 (17)	0.38126 (8)	0.01460 (18)	
H3	0.078164	0.685935	0.360157	0.018*	
C2	0.31334 (18)	0.70431 (17)	0.48778 (8)	0.01512 (18)	
H2	0.203579	0.661662	0.539888	0.018*	
C1	0.54914 (18)	0.75198 (15)	0.51796 (8)	0.01165 (16)	
C6	0.71054 (18)	0.81524 (16)	0.44154 (8)	0.01307 (17)	
H6	0.871163	0.847484	0.462431	0.016*	
C5	0.63753 (17)	0.83133 (16)	0.33499 (8)	0.01266 (17)	
H5	0.747219	0.874444	0.282876	0.015*	
C7	0.62381 (19)	0.73240 (15)	0.63257 (8)	0.01397 (18)	

### Tetraaquabis(4-carboxybenzenesulfonato)copper(II) dihydrate (I) Atomic displacement parameters (Å^2^)

**Table:**

	*U*^11^	*U*^22^	*U*^33^	*U*^12^	*U*^13^	*U*^23^
Cu1	0.00874 (8)	0.01025 (8)	0.00916 (8)	0.00255 (6)	−0.00025 (5)	0.00210 (5)
S1	0.00890 (10)	0.00930 (10)	0.00886 (10)	0.00283 (8)	0.00080 (7)	0.00029 (7)
O5	0.0117 (3)	0.0130 (3)	0.0134 (3)	0.0022 (3)	0.0014 (2)	0.0042 (3)
O4	0.0167 (3)	0.0119 (3)	0.0134 (3)	0.0047 (3)	0.0024 (3)	−0.0024 (3)
O3	0.0105 (3)	0.0158 (3)	0.0156 (3)	0.0061 (3)	−0.0001 (3)	−0.0002 (3)
O2	0.0238 (4)	0.0293 (5)	0.0126 (4)	0.0088 (4)	0.0055 (3)	0.0080 (3)
O1	0.0178 (4)	0.0267 (4)	0.0113 (3)	0.0043 (3)	−0.0021 (3)	0.0023 (3)
O6	0.0097 (3)	0.0108 (3)	0.0145 (3)	0.0038 (2)	0.0026 (2)	0.0036 (2)
O7	0.0108 (3)	0.0146 (3)	0.0100 (3)	0.0025 (3)	0.0006 (2)	0.0024 (2)
O8	0.0109 (3)	0.0127 (3)	0.0188 (4)	0.0033 (3)	0.0013 (3)	0.0041 (3)
C4	0.0110 (4)	0.0106 (4)	0.0096 (4)	0.0038 (3)	0.0014 (3)	0.0006 (3)
C3	0.0112 (4)	0.0203 (5)	0.0127 (4)	0.0059 (4)	0.0030 (3)	0.0029 (4)
C2	0.0137 (4)	0.0196 (5)	0.0119 (4)	0.0052 (4)	0.0044 (3)	0.0039 (4)
C1	0.0143 (4)	0.0112 (4)	0.0094 (4)	0.0045 (3)	0.0016 (3)	0.0010 (3)
C6	0.0112 (4)	0.0158 (4)	0.0114 (4)	0.0039 (3)	0.0009 (3)	0.0004 (3)
C5	0.0107 (4)	0.0158 (4)	0.0106 (4)	0.0033 (3)	0.0023 (3)	0.0007 (3)
C7	0.0189 (5)	0.0123 (4)	0.0109 (4)	0.0059 (4)	0.0011 (3)	0.0011 (3)

### Tetraaquabis(4-carboxybenzenesulfonato)copper(II) dihydrate (I) Geometric parameters (Å, º)

**Table:**

Cu1—O6	1.9520 (7)	O7—H7A	0.831 (9)
Cu1—O6^i^	1.9521 (7)	O7—H7B	0.840 (9)
Cu1—O7	1.9743 (7)	O8—H8B	0.822 (9)
Cu1—O7^i^	1.9743 (7)	O8—H8A	0.832 (9)
Cu1—O4	2.3934 (8)	C4—C5	1.3916 (14)
Cu1—O4^i^	2.3934 (8)	C4—C3	1.3934 (14)
S1—O4	1.4593 (8)	C3—C2	1.3906 (15)
S1—O5	1.4596 (8)	C3—H3	0.9500
S1—O3	1.4607 (8)	C2—C1	1.3931 (15)
S1—C4	1.7760 (10)	C2—H2	0.9500
O2—C7	1.2152 (13)	C1—C6	1.3954 (14)
O1—C7	1.3216 (14)	C1—C7	1.4922 (14)
O1—H1	0.78 (2)	C6—C5	1.3901 (14)
O6—H6B	0.814 (9)	C6—H6	0.9500
O6—H6A	0.813 (9)	C5—H5	0.9500
			
O6—Cu1—O6^i^	180.00 (3)	Cu1—O7—H7A	111.5 (12)
O6—Cu1—O7	90.38 (3)	Cu1—O7—H7B	112.3 (12)
O6^i^—Cu1—O7	89.62 (3)	H7A—O7—H7B	108.6 (16)
O6—Cu1—O7^i^	89.62 (3)	H8B—O8—H8A	104.9 (17)
O6^i^—Cu1—O7^i^	90.38 (3)	C5—C4—C3	121.01 (9)
O7—Cu1—O7^i^	180.0	C5—C4—S1	119.92 (7)
O6—Cu1—O4	87.38 (3)	C3—C4—S1	119.05 (8)
O6^i^—Cu1—O4	92.62 (3)	C2—C3—C4	119.54 (9)
O7—Cu1—O4	90.00 (3)	C2—C3—H3	120.2
O7^i^—Cu1—O4	90.01 (3)	C4—C3—H3	120.2
O6—Cu1—O4^i^	92.62 (3)	C3—C2—C1	119.90 (9)
O6^i^—Cu1—O4^i^	87.38 (3)	C3—C2—H2	120.0
O7—Cu1—O4^i^	90.01 (3)	C1—C2—H2	120.0
O7^i^—Cu1—O4^i^	89.99 (3)	C2—C1—C6	120.09 (9)
O4—Cu1—O4^i^	180.0	C2—C1—C7	118.74 (9)
O4—S1—O5	113.02 (5)	C6—C1—C7	121.17 (9)
O4—S1—O3	112.56 (5)	C5—C6—C1	120.35 (9)
O5—S1—O3	112.31 (5)	C5—C6—H6	119.8
O4—S1—C4	106.04 (5)	C1—C6—H6	119.8
O5—S1—C4	106.46 (5)	C6—C5—C4	119.11 (9)
O3—S1—C4	105.77 (5)	C6—C5—H5	120.4
S1—O4—Cu1	134.82 (5)	C4—C5—H5	120.4
C7—O1—H1	109.5	O2—C7—O1	124.44 (10)
Cu1—O6—H6B	116.7 (12)	O2—C7—C1	122.18 (10)
Cu1—O6—H6A	117.3 (12)	O1—C7—C1	113.39 (9)
H6B—O6—H6A	106.8 (16)		

Symmetry code: (i) −*x*+1, −*y*+1, −*z*.

### Tetraaquabis(4-carboxybenzenesulfonato)copper(II) dihydrate (I) Hydrogen-bond geometry (Å, º)

**Table:**

*D*—H···*A*	*D*—H	H···*A*	*D*···*A*	*D*—H···*A*
O1—H1···O8^ii^	0.78	1.94	2.6979 (12)	164
O6—H6*B*···O5^iii^	0.81 (1)	1.97 (1)	2.7738 (11)	172 (2)
O6—H6*A*···O8^iv^	0.81 (1)	1.88 (1)	2.6872 (11)	172 (2)
O7—H7*A*···O2^v^	0.83 (1)	1.86 (1)	2.6845 (11)	171 (2)
O7—H7*B*···O3^vi^	0.84 (1)	1.84 (1)	2.6672 (11)	169 (2)
O8—H8*B*···O7	0.82 (1)	2.17 (1)	2.9255 (11)	153 (2)
O8—H8*A*···O5^iii^	0.83 (1)	1.99 (1)	2.7984 (11)	165 (2)

Symmetry codes: (ii) −*x*+2, −*y*+1, −*z*+1; (iii) *x*, *y*−1, *z*; (iv) *x*−1, *y*, *z*; (v) −*x*+1, −*y*+1, −*z*+1; (vi) *x*+1, *y*, *z*.

### Hexaaquacopper(II) 4-carboxybenzenesulfonate (II) Crystal data

**Table:**

[Cu(H_2_O)_6_](C_7_H_5_O_5_S)_2_	*Z* = 1
*M**_r_* = 573.97	*F*(000) = 295
Triclinic, *P*1	*D*_x_ = 1.822 Mg m^−^^3^
*a* = 6.4380 (13) Å	Mo *K*α radiation, λ = 0.71073 Å
*b* = 7.2431 (14) Å	Cell parameters from 4072 reflections
*c* = 12.088 (2) Å	θ = 3.3–33.0°
α = 72.60 (3)°	µ = 1.33 mm^−^^1^
β = 77.20 (3)°	*T* = 130 K
γ = 82.13 (3)°	Thin plate, light blue
*V* = 523.0 (2) Å^3^	0.21 × 0.08 × 0.02 mm

### Hexaaquacopper(II) 4-carboxybenzenesulfonate (II) Data collection

**Table:**

Bruker APEXII CCD diffractometer	3370 reflections with *I* > 2σ(*I*)
Radiation source: sealed tube	*R*_int_ = 0.045
φ and ω scans	θ_max_ = 33.0°, θ_min_ = 3.0°
Absorption correction: multi-scan (*TWINABS*; Sheldrick, 1996)	*h* = −9→9
*T*_min_ = 0.585, *T*_max_ = 0.747	*k* = −10→10
3738 measured reflections	*l* = 0→18
3738 independent reflections	

### Hexaaquacopper(II) 4-carboxybenzenesulfonate (II) Refinement

**Table:**

Refinement on *F*^2^	Primary atom site location: structure-invariant direct methods
Least-squares matrix: full	Secondary atom site location: difference Fourier map
*R*[*F*^2^ > 2σ(*F*^2^)] = 0.059	Hydrogen site location: mixed
*wR*(*F*^2^) = 0.139	H atoms treated by a mixture of independent and constrained refinement
*S* = 1.18	*w* = 1/[σ^2^(*F*_o_^2^) + (0.0332*P*)^2^ + 2.049*P*] where *P* = (*F*_o_^2^ + 2*F*_c_^2^)/3
3738 reflections	(Δ/σ)_max_ = 0.001
169 parameters	Δρ_max_ = 0.84 e Å^−^^3^
18 restraints	Δρ_min_ = −1.19 e Å^−^^3^

### Hexaaquacopper(II) 4-carboxybenzenesulfonate (II) Special details

**Table:**

Geometry. All esds (except the esd in the dihedral angle between two l.s. planes) are estimated using the full covariance matrix. The cell esds are taken into account individually in the estimation of esds in distances, angles and torsion angles; correlations between esds in cell parameters are only used when they are defined by crystal symmetry. An approximate (isotropic) treatment of cell esds is used for estimating esds involving l.s. planes.
Refinement. Refined as a 2-component twin. BASF refines to: 0.47690

### Hexaaquacopper(II) 4-carboxybenzenesulfonate (II) Fractional atomic coordinates and isotropic or equivalent isotropic displacement parameters (Å^2^)

**Table:**

	*x*	*y*	*z*	*U*_iso_*/*U*_eq_	Occ. (<1)
Cu1	0.5000	1.0000	0.0000	0.00922 (14)	
S1	0.89992 (13)	0.53760 (18)	0.82865 (7)	0.01095 (17)	
O5	1.0191 (5)	0.3517 (5)	0.8217 (3)	0.0173 (6)	
O4	0.7159 (4)	0.5122 (5)	0.92630 (19)	0.0115 (4)	
O3	1.0340 (5)	0.6825 (4)	0.8291 (3)	0.0162 (7)	
O2	0.6930 (5)	0.9432 (6)	0.2844 (3)	0.0241 (7)	
O1	0.3772 (5)	0.8181 (7)	0.3778 (3)	0.0256 (7)	
H1	0.333 (10)	0.874 (12)	0.313 (6)	0.038*	
O6	0.6358 (5)	0.7475 (5)	0.0720 (3)	0.0135 (6)	
H61	0.744 (6)	0.733 (8)	0.102 (4)	0.020*	
H62	0.639 (8)	0.656 (6)	0.042 (5)	0.020*	
O7	0.6741 (5)	1.1291 (5)	0.0619 (3)	0.0145 (6)	
H71	0.700 (9)	1.245 (4)	0.035 (4)	0.022*	
H72	0.706 (9)	1.082 (7)	0.128 (3)	0.022*	
O8	0.2112 (4)	0.9557 (5)	0.1819 (2)	0.0128 (5)	
H81	0.124 (7)	1.056 (5)	0.175 (5)	0.019*	
H82	0.143 (8)	0.863 (6)	0.186 (5)	0.019*	
C4	0.7954 (6)	0.6287 (8)	0.6968 (3)	0.0168 (8)	
C3A	0.6064 (13)	0.5844 (14)	0.6900 (7)	0.0106 (13)*	0.5
H3A	0.5218	0.5095	0.7567	0.013*	0.5
C2A	0.5338 (14)	0.6495 (14)	0.5834 (7)	0.0134 (15)*	0.5
H2A	0.4059	0.6102	0.5777	0.016*	0.5
C3B	0.5727 (13)	0.6505 (16)	0.7017 (7)	0.0158 (14)*	0.5
H3B	0.4774	0.6196	0.7735	0.019*	0.5
C2B	0.5006 (14)	0.7198 (15)	0.5951 (7)	0.0173 (16)*	0.5
H2B	0.3551	0.7325	0.5947	0.021*	0.5
C1	0.6503 (6)	0.7712 (6)	0.4869 (3)	0.0155 (8)	
C6A	0.8529 (12)	0.8324 (15)	0.4945 (6)	0.0144 (13)*	0.5
H6A	0.9325	0.9140	0.4288	0.017*	0.5
C5A	0.9247 (12)	0.7684 (15)	0.5998 (6)	0.0156 (13)*	0.5
H5A	1.0479	0.8108	0.6093	0.019*	0.5
C6B	0.8561 (12)	0.7127 (16)	0.4865 (7)	0.0174 (13)*	0.5
H6B	0.9507	0.7289	0.4146	0.021*	0.5
C5B	0.9332 (13)	0.6284 (14)	0.5903 (7)	0.0183 (15)*	0.5
H5B	1.0727	0.5734	0.5887	0.022*	0.5
C7	0.5754 (7)	0.8560 (6)	0.3725 (4)	0.0155 (8)	

### Hexaaquacopper(II) 4-carboxybenzenesulfonate (II) Atomic displacement parameters (Å^2^)

**Table:**

	*U*^11^	*U*^22^	*U*^33^	*U*^12^	*U*^13^	*U*^23^
Cu1	0.0126 (3)	0.0084 (3)	0.0077 (2)	−0.0002 (3)	−0.00533 (19)	−0.0016 (3)
S1	0.0104 (4)	0.0135 (4)	0.0097 (3)	0.0005 (4)	−0.0037 (3)	−0.0037 (4)
O5	0.0155 (14)	0.0217 (17)	0.0183 (14)	0.0048 (12)	−0.0086 (11)	−0.0099 (12)
O4	0.0124 (11)	0.0130 (13)	0.0080 (9)	−0.0008 (13)	−0.0021 (8)	−0.0013 (12)
O3	0.0147 (14)	0.0132 (17)	0.0231 (15)	−0.0041 (11)	−0.0030 (11)	−0.0078 (11)
O2	0.0245 (15)	0.0318 (19)	0.0118 (12)	−0.0011 (15)	−0.0060 (11)	0.0017 (14)
O1	0.0266 (15)	0.037 (2)	0.0147 (12)	−0.0015 (18)	−0.0124 (11)	−0.0029 (17)
O6	0.0178 (14)	0.0099 (14)	0.0148 (13)	0.0014 (11)	−0.0073 (11)	−0.0045 (11)
O7	0.0203 (15)	0.0140 (15)	0.0115 (12)	−0.0077 (11)	−0.0087 (11)	−0.0001 (11)
O8	0.0123 (11)	0.0131 (14)	0.0144 (10)	−0.0003 (12)	−0.0044 (9)	−0.0048 (12)
C4	0.0129 (15)	0.029 (2)	0.0088 (13)	0.0015 (18)	−0.0038 (11)	−0.0056 (18)
C1	0.0185 (18)	0.019 (2)	0.0090 (14)	0.0053 (14)	−0.0045 (12)	−0.0051 (13)
C7	0.026 (2)	0.0118 (17)	0.0115 (15)	0.0016 (14)	−0.0075 (14)	−0.0058 (13)

### Hexaaquacopper(II) 4-carboxybenzenesulfonate (II) Geometric parameters (Å, º)

**Table:**

Cu1—O7	1.941 (3)	C4—C5B	1.393 (9)
Cu1—O7^i^	1.941 (3)	C4—C3B	1.411 (9)
Cu1—O6^i^	1.953 (3)	C4—C5A	1.476 (9)
Cu1—O6	1.953 (3)	C3A—C2A	1.395 (11)
Cu1—O8	2.515 (3)	C3A—H3A	0.9300
Cu1—O8^i^	2.515 (3)	C2A—C1	1.370 (9)
S1—O3	1.449 (3)	C2A—H2A	0.9300
S1—O4	1.463 (2)	C3B—C2B	1.394 (11)
S1—O5	1.472 (4)	C3B—H3B	0.9300
S1—C4	1.776 (4)	C2B—C1	1.424 (9)
O2—C7	1.216 (5)	C2B—H2B	0.9300
O1—C7	1.325 (6)	C1—C6B	1.334 (9)
O1—H1	0.86 (7)	C1—C6A	1.464 (9)
O6—H61	0.84 (2)	C1—C7	1.493 (5)
O6—H62	0.84 (2)	C6A—C5A	1.377 (10)
O7—H71	0.83 (2)	C6A—H6A	0.9300
O7—H72	0.83 (2)	C5A—H5A	0.9300
O8—H81	0.85 (2)	C6B—C5B	1.388 (11)
O8—H82	0.84 (2)	C6B—H6B	0.9300
C4—C3A	1.326 (9)	C5B—H5B	0.9300
			
O7—Cu1—O7^i^	180.0	C4—C3A—H3A	119.8
O7—Cu1—O6^i^	89.23 (12)	C2A—C3A—H3A	119.8
O7^i^—Cu1—O6^i^	90.77 (12)	C1—C2A—C3A	120.3 (7)
O7—Cu1—O6	90.77 (12)	C1—C2A—H2A	119.9
O7^i^—Cu1—O6	89.23 (12)	C3A—C2A—H2A	119.9
O6^i^—Cu1—O6	180.0	C2B—C3B—C4	117.6 (7)
O7—Cu1—O8	92.88 (12)	C2B—C3B—H3B	121.2
O7^i^—Cu1—O8	87.12 (12)	C4—C3B—H3B	121.2
O8—Cu1—O8^i^	180.0	C3B—C2B—C1	119.8 (7)
O6—Cu1—O8	89.03 (12)	C3B—C2B—H2B	120.1
O6—Cu1—O8^i^	90.97 (12)	C1—C2B—H2B	120.1
O3—S1—O4	112.4 (2)	C6B—C1—C2B	118.9 (6)
O3—S1—O5	113.31 (17)	C2A—C1—C6A	120.3 (5)
O4—S1—O5	112.1 (2)	C6B—C1—C7	119.6 (5)
O3—S1—C4	106.3 (2)	C2A—C1—C7	123.0 (5)
O4—S1—C4	106.37 (16)	C2B—C1—C7	120.5 (5)
O5—S1—C4	105.8 (2)	C6A—C1—C7	116.6 (4)
C7—O1—H1	112 (4)	C5A—C6A—C1	119.5 (7)
Cu1—O6—H61	124 (4)	C5A—C6A—H6A	120.2
Cu1—O6—H62	119 (4)	C1—C6A—H6A	120.2
H61—O6—H62	108 (4)	C6A—C5A—C4	116.4 (7)
Cu1—O7—H71	125 (4)	C6A—C5A—H5A	121.8
Cu1—O7—H72	123 (3)	C4—C5A—H5A	121.8
H71—O7—H72	111 (4)	C1—C6B—C5B	122.0 (7)
H81—O8—H82	107 (3)	C1—C6B—H6B	119.0
C5B—C4—C3B	119.5 (5)	C5B—C6B—H6B	119.0
C3A—C4—C5A	122.7 (5)	C6B—C5B—C4	118.0 (7)
C3A—C4—S1	121.7 (4)	C6B—C5B—H5B	121.0
C5B—C4—S1	118.0 (4)	C4—C5B—H5B	121.0
C3B—C4—S1	120.3 (4)	O2—C7—O1	125.5 (4)
C5A—C4—S1	115.4 (4)	O2—C7—C1	121.3 (4)
C4—C3A—C2A	120.3 (7)	O1—C7—C1	113.1 (4)

Symmetry code: (i) −*x*+1, −*y*+2, −*z*.

### Hexaaquacopper(II) 4-carboxybenzenesulfonate (II) Hydrogen-bond geometry (Å, º)

**Table:**

*D*—H···*A*	*D*—H	H···*A*	*D*···*A*	*D*—H···*A*
O1—H1···O8	0.86 (7)	1.83 (7)	2.677 (4)	170 (7)
O6—H61···O5^ii^	0.84 (2)	1.89 (3)	2.717 (4)	169 (5)
O6—H62···O4^iii^	0.84 (2)	1.93 (3)	2.725 (5)	158 (5)
O7—H71···O4^iv^	0.83 (2)	1.99 (3)	2.784 (5)	160 (5)
O7—H72···O2	0.83 (2)	1.84 (3)	2.645 (4)	161 (5)
O8—H81···O3^v^	0.85 (2)	2.02 (3)	2.851 (5)	167 (5)
O8—H82···O5^vi^	0.84 (2)	2.02 (3)	2.854 (5)	175 (5)

Symmetry codes: (ii) −*x*+2, −*y*+1, −*z*+1; (iii) *x*, *y*, *z*−1; (iv) *x*, *y*+1, *z*−1; (v) −*x*+1, −*y*+2, −*z*+1; (vi) −*x*+1, −*y*+1, −*z*+1.

### Silver potassium 4-carboxybenzenesulfonate (III) Crystal data

**Table:**

[Ag_0.69_K_0.31_](C_7_H_5_O_5_S)	*F*(000) = 1130.6
*M**_r_* = 287.72	*D*_x_ = 2.368 Mg m^−^^3^
Monoclinic, *C*2/*c*	Mo *K*α radiation, λ = 0.71073 Å
*a* = 19.436 (3) Å	Cell parameters from 7014 reflections
*b* = 15.644 (3) Å	θ = 2.6–30.4°
*c* = 5.3355 (9) Å	µ = 2.17 mm^−^^1^
β = 95.651 (2)°	*T* = 120 K
*V* = 1614.4 (5) Å^3^	Needle, colorless
*Z* = 8	0.16 × 0.06 × 0.03 mm

### Silver potassium 4-carboxybenzenesulfonate (III) Data collection

**Table:**

Bruker APEXII CCD diffractometer	2435 independent reflections
Radiation source: fine-focus sealed tube	2223 reflections with *I* > 2σ(*I*)
Curved graphite crystal monochromator	*R*_int_ = 0.020
ω scans	θ_max_ = 30.4°, θ_min_ = 1.7°
Absorption correction: multi-scan (SADABS; Krause *et al.*, 2015)	*h* = −27→27
*T*_min_ = 0.572, *T*_max_ = 0.648	*k* = −22→22
12751 measured reflections	*l* = −7→7

### Silver potassium 4-carboxybenzenesulfonate (III) Refinement

**Table:**

Refinement on *F*^2^	Primary atom site location: dual
Least-squares matrix: full	Secondary atom site location: difference Fourier map
*R*[*F*^2^ > 2σ(*F*^2^)] = 0.017	Hydrogen site location: inferred from neighbouring sites
*wR*(*F*^2^) = 0.043	H atoms treated by a mixture of independent and constrained refinement
*S* = 1.08	*w* = 1/[σ^2^(*F*_o_^2^) + (0.021*P*)^2^ + 1.6847*P*] where *P* = (*F*_o_^2^ + 2*F*_c_^2^)/3
2435 reflections	(Δ/σ)_max_ = 0.001
135 parameters	Δρ_max_ = 0.51 e Å^−^^3^
0 restraints	Δρ_min_ = −0.36 e Å^−^^3^

### Silver potassium 4-carboxybenzenesulfonate (III) Special details

**Table:**

Geometry. All esds (except the esd in the dihedral angle between two l.s. planes) are estimated using the full covariance matrix. The cell esds are taken into account individually in the estimation of esds in distances, angles and torsion angles; correlations between esds in cell parameters are only used when they are defined by crystal symmetry. An approximate (isotropic) treatment of cell esds is used for estimating esds involving l.s. planes.

### Silver potassium 4-carboxybenzenesulfonate (III) Fractional atomic coordinates and isotropic or equivalent isotropic displacement parameters (Å^2^)

**Table:**

	*x*	*y*	*z*	*U*_iso_*/*U*_eq_	Occ. (<1)
Ag1	0.500000	0.54234 (2)	0.750000	0.01208 (5)	
Ag2	0.500000	0.2558 (3)	0.250000	0.0138 (5)	0.38
K2	0.500000	0.2420 (5)	0.250000	0.0119 (6)	0.62
S1	0.39857 (2)	0.35832 (2)	0.72451 (6)	0.00889 (7)	
O1	0.08310 (6)	0.41836 (8)	0.1298 (2)	0.0193 (2)	
H1	0.0452 (12)	0.4128 (14)	0.0891 (18)	0.029*	
O2	0.05037 (5)	0.38234 (8)	0.5066 (2)	0.0210 (2)	
O3	0.43544 (5)	0.35558 (7)	0.4987 (2)	0.0145 (2)	
O4	0.41399 (5)	0.43608 (7)	0.8736 (2)	0.0130 (2)	
O5	0.40550 (5)	0.28066 (7)	0.8741 (2)	0.0155 (2)	
C1	0.17074 (7)	0.38502 (9)	0.4510 (3)	0.0130 (3)	
C2	0.22060 (7)	0.41777 (9)	0.3068 (3)	0.0140 (3)	
H2	0.206693	0.445860	0.152193	0.017*	
C3	0.29056 (7)	0.40970 (9)	0.3872 (3)	0.0127 (3)	
H3	0.324600	0.432562	0.289897	0.015*	
C4	0.30975 (7)	0.36746 (9)	0.6130 (3)	0.0100 (2)	
C5	0.26050 (7)	0.33392 (10)	0.7577 (3)	0.0136 (3)	
H5	0.274541	0.304689	0.910281	0.016*	
C6	0.19057 (7)	0.34337 (10)	0.6781 (3)	0.0154 (3)	
H6	0.156577	0.321642	0.777569	0.018*	
C7	0.09604 (7)	0.39454 (10)	0.3672 (3)	0.0159 (3)	

### Silver potassium 4-carboxybenzenesulfonate (III) Atomic displacement parameters (Å^2^)

**Table:**

	*U*^11^	*U*^22^	*U*^33^	*U*^12^	*U*^13^	*U*^23^
Ag1	0.01209 (7)	0.01398 (8)	0.01001 (7)	0.000	0.00026 (5)	0.000
Ag2	0.0148 (4)	0.0146 (12)	0.0123 (4)	0.000	0.0023 (3)	0.000
K2	0.0102 (6)	0.014 (2)	0.0113 (6)	0.000	0.0009 (4)	0.000
S1	0.00656 (13)	0.01002 (15)	0.01008 (15)	−0.00029 (10)	0.00077 (11)	−0.00019 (11)
O1	0.0113 (5)	0.0227 (6)	0.0223 (6)	−0.0008 (4)	−0.0068 (4)	0.0043 (4)
O2	0.0095 (5)	0.0330 (6)	0.0199 (5)	0.0031 (4)	−0.0021 (4)	−0.0051 (5)
O3	0.0110 (5)	0.0195 (5)	0.0140 (5)	0.0004 (4)	0.0053 (4)	−0.0017 (4)
O4	0.0104 (4)	0.0130 (5)	0.0150 (5)	−0.0009 (4)	−0.0016 (4)	−0.0036 (4)
O5	0.0119 (5)	0.0137 (5)	0.0204 (5)	0.0003 (4)	−0.0006 (4)	0.0056 (4)
C1	0.0093 (6)	0.0142 (6)	0.0148 (6)	0.0005 (5)	−0.0029 (5)	−0.0039 (5)
C2	0.0132 (6)	0.0141 (6)	0.0139 (6)	−0.0001 (5)	−0.0028 (5)	0.0011 (5)
C3	0.0118 (6)	0.0136 (6)	0.0125 (6)	−0.0018 (5)	0.0004 (5)	0.0011 (5)
C4	0.0077 (5)	0.0107 (6)	0.0113 (6)	−0.0002 (4)	−0.0003 (5)	−0.0019 (5)
C5	0.0091 (6)	0.0192 (7)	0.0125 (6)	−0.0004 (5)	0.0005 (5)	0.0025 (5)
C6	0.0088 (6)	0.0228 (7)	0.0146 (6)	−0.0018 (5)	0.0009 (5)	0.0004 (5)
C7	0.0115 (6)	0.0151 (7)	0.0200 (7)	0.0015 (5)	−0.0047 (5)	−0.0036 (5)

### Silver potassium 4-carboxybenzenesulfonate (III) Geometric parameters (Å, º)

**Table:**

Ag1—O4	2.4919 (11)	S1—O5	1.4525 (11)
Ag1—O4^i^	2.4920 (11)	S1—O3	1.4616 (11)
Ag1—O3^ii^	2.4928 (11)	S1—O4	1.4682 (11)
Ag1—O3^iii^	2.4928 (11)	S1—C4	1.7756 (14)
Ag1—O4^iv^	2.5061 (10)	O1—C7	1.3205 (19)
Ag1—O4^v^	2.5061 (10)	O1—H1	0.75 (2)
Ag1—Ag1^iii^	2.9785 (4)	O2—C7	1.2282 (19)
Ag1—Ag1^iv^	2.9785 (4)	C1—C2	1.393 (2)
Ag1—Ag2^iii^	3.158 (5)	C1—C6	1.396 (2)
Ag1—K2^iii^	3.373 (8)	C1—C7	1.4840 (19)
Ag2—O3^vi^	2.470 (3)	C2—C3	1.3906 (19)
Ag2—O3	2.470 (3)	C2—H2	0.9500
K2—O2^vii^	2.584 (6)	C3—C4	1.3925 (19)
K2—O2^viii^	2.584 (6)	C3—H3	0.9500
K2—O3^vi^	2.611 (6)	C4—C5	1.3904 (19)
K2—O3	2.612 (6)	C5—C6	1.3913 (19)
K2—O5^i^	2.653 (2)	C5—H5	0.9500
K2—O5^ix^	2.653 (2)	C6—H6	0.9500
			
O4—Ag1—O4^i^	96.32 (5)	O3—S1—C4	105.40 (7)
O4—Ag1—O3^ii^	84.29 (4)	O4—S1—C4	104.75 (6)
O4^i^—Ag1—O3^ii^	162.59 (4)	C7—O1—H1	109.5
O4—Ag1—O3^iii^	162.59 (4)	C7—O2—K2^viii^	138.90 (15)
O4^i^—Ag1—O3^iii^	84.29 (4)	S1—O3—Ag2	141.04 (10)
O3^ii^—Ag1—O3^iii^	100.33 (5)	S1—O3—Ag1^iii^	137.17 (6)
O4—Ag1—O4^iv^	106.84 (3)	Ag2—O3—Ag1^iii^	79.04 (9)
O4^i^—Ag1—O4^iv^	83.68 (4)	S1—O3—K2	137.55 (14)
O3^ii^—Ag1—O4^iv^	79.52 (4)	Ag1^iii^—O3—K2	82.70 (13)
O3^iii^—Ag1—O4^iv^	90.53 (4)	S1—O4—Ag1	121.05 (6)
O4—Ag1—O4^v^	83.68 (4)	S1—O4—Ag1^iv^	129.07 (6)
O4^i^—Ag1—O4^v^	106.84 (3)	Ag1—O4—Ag1^iv^	73.16 (3)
O3^ii^—Ag1—O4^v^	90.53 (4)	S1—O5—K2^x^	128.74 (17)
O3^iii^—Ag1—O4^v^	79.52 (4)	C2—C1—C6	120.23 (13)
O4^iv^—Ag1—O4^v^	164.51 (5)	C2—C1—C7	120.64 (13)
O3^vi^—Ag2—O3	101.60 (17)	C6—C1—C7	119.14 (13)
O2^vii^—K2—O2^viii^	82.3 (2)	C3—C2—C1	120.56 (13)
O2^vii^—K2—O3^vi^	91.83 (4)	C3—C2—H2	119.7
O2^viii^—K2—O3^vi^	172.8 (2)	C1—C2—H2	119.7
O2^vii^—K2—O3	172.8 (2)	C2—C3—C4	118.71 (13)
O2^viii^—K2—O3	91.84 (4)	C2—C3—H3	120.6
O3^vi^—K2—O3	94.3 (3)	C4—C3—H3	120.6
O2^vii^—K2—O5^i^	106.44 (13)	C5—C4—C3	121.28 (13)
O2^viii^—K2—O5^i^	93.44 (10)	C5—C4—S1	118.90 (11)
O3^vi^—K2—O5^i^	84.13 (15)	C3—C4—S1	119.80 (10)
O3—K2—O5^i^	78.01 (13)	C4—C5—C6	119.71 (13)
O2^vii^—K2—O5^ix^	93.44 (10)	C4—C5—H5	120.1
O2^viii^—K2—O5^ix^	106.44 (13)	C6—C5—H5	120.1
O3^vi^—K2—O5^ix^	78.01 (13)	C5—C6—C1	119.50 (13)
O3—K2—O5^ix^	84.13 (15)	C5—C6—H6	120.3
O5^i^—K2—O5^ix^	153.7 (3)	C1—C6—H6	120.3
O5—S1—O3	113.70 (6)	O2—C7—O1	122.99 (13)
O5—S1—O4	113.11 (7)	O2—C7—C1	123.07 (14)
O3—S1—O4	112.35 (6)	O1—C7—C1	113.94 (13)
O5—S1—C4	106.62 (6)		

Symmetry codes: (i) −*x*+1, *y*, −*z*+3/2; (ii) *x*, −*y*+1, *z*+1/2; (iii) −*x*+1, −*y*+1, −*z*+1; (iv) −*x*+1, −*y*+1, −*z*+2; (v) *x*, −*y*+1, *z*−1/2; (vi) −*x*+1, *y*, −*z*+1/2; (vii) *x*+1/2, −*y*+1/2, *z*−1/2; (viii) −*x*+1/2, −*y*+1/2, −*z*+1; (ix) *x*, *y*, *z*−1; (x) *x*, *y*, *z*+1.

### Silver potassium 4-carboxybenzenesulfonate (III) Hydrogen-bond geometry (Å, º)

**Table:**

*D*—H···*A*	*D*—H	H···*A*	*D*···*A*	*D*—H···*A*
O1—H1···O2^xi^	0.75	1.94	2.6841 (16)	172

Symmetry code: (xi) −*x*, *y*, −*z*+1/2.

### Potassium silver 4-carboxybenzenesulfonate salt dihydrate (IV) Crystal data

**Table:**

[Ag_0.20_K_0.80_](C_7_H_5_O_5_S)·2H_2_O	*F*(000) = 590.4
*M**_r_* = 290.06	*D*_x_ = 1.813 Mg m^−^^3^
Monoclinic, *P*2_1_/*c*	Mo *K*α radiation, λ = 0.71073 Å
*a* = 12.8018 (7) Å	Cell parameters from 8713 reflections
*b* = 9.9170 (6) Å	θ = 2.6–30.6°
*c* = 8.4013 (5) Å	µ = 0.99 mm^−^^1^
β = 94.747 (1)°	*T* = 120 K
*V* = 1062.93 (11) Å^3^	Plate, colorless
*Z* = 4	0.23 × 0.17 × 0.07 mm

### Potassium silver 4-carboxybenzenesulfonate salt dihydrate (IV) Data collection

**Table:**

Bruker APEXII CCD diffractometer	3255 independent reflections
Radiation source: fine-focus sealed tube	2831 reflections with *I* > 2σ(*I*)
Curved graphite crystal monochromator	*R*_int_ = 0.023
ω scans	θ_max_ = 30.6°, θ_min_ = 1.6°
Absorption correction: multi-scan (SADABS; Krause *et al.*, 2015)	*h* = −18→18
*T*_min_ = 0.666, *T*_max_ = 0.746	*k* = −14→14
16575 measured reflections	*l* = −12→12

### Potassium silver 4-carboxybenzenesulfonate salt dihydrate (IV) Refinement

**Table:**

Refinement on *F*^2^	Primary atom site location: dual
Least-squares matrix: full	Secondary atom site location: difference Fourier map
*R*[*F*^2^ > 2σ(*F*^2^)] = 0.025	Hydrogen site location: mixed
*wR*(*F*^2^) = 0.063	H atoms treated by a mixture of independent and constrained refinement
*S* = 1.08	*w* = 1/[σ^2^(*F*_o_^2^) + (0.0273*P*)^2^ + 0.5631*P*] where *P* = (*F*_o_^2^ + 2*F*_c_^2^)/3
3255 reflections	(Δ/σ)_max_ = 0.003
171 parameters	Δρ_max_ = 0.44 e Å^−^^3^
4 restraints	Δρ_min_ = −0.54 e Å^−^^3^

### Potassium silver 4-carboxybenzenesulfonate salt dihydrate (IV) Special details

**Table:**

Geometry. All esds (except the esd in the dihedral angle between two l.s. planes) are estimated using the full covariance matrix. The cell esds are taken into account individually in the estimation of esds in distances, angles and torsion angles; correlations between esds in cell parameters are only used when they are defined by crystal symmetry. An approximate (isotropic) treatment of cell esds is used for estimating esds involving l.s. planes.

### Potassium silver 4-carboxybenzenesulfonate salt dihydrate (IV) Fractional atomic coordinates and isotropic or equivalent isotropic displacement parameters (Å^2^)

**Table:**

	*x*	*y*	*z*	*U*_iso_*/*U*_eq_	Occ. (<1)
K1	0.95958 (2)	0.23895 (2)	0.86257 (3)	0.02002 (6)	0.8
Ag1	0.95958 (2)	0.23895 (2)	0.86257 (3)	0.02002 (6)	0.2
S1	0.14513 (2)	0.39457 (3)	0.64063 (4)	0.01598 (7)	
O1	0.66150 (8)	0.46300 (11)	0.81388 (14)	0.0255 (2)	
H1	0.7195 (16)	0.4598 (16)	0.8542 (19)	0.038*	
O2	0.63265 (8)	0.30700 (11)	0.99903 (14)	0.0273 (2)	
O3A	0.14016 (19)	0.4401 (3)	0.4747 (3)	0.0212 (4)	0.8
O4A	0.0969 (2)	0.4883 (3)	0.7438 (3)	0.0233 (5)	0.8
O5A	0.1049 (2)	0.2565 (3)	0.6567 (3)	0.0267 (6)	0.8
O3B	0.1341 (10)	0.4662 (10)	0.4939 (15)	0.021 (2)*	0.2
O4B	0.0914 (9)	0.4677 (11)	0.7709 (13)	0.0120 (18)*	0.2
O5B	0.1040 (9)	0.2604 (12)	0.6199 (11)	0.0091 (16)*	0.2
O6	0.85970 (8)	0.48285 (10)	0.92292 (13)	0.0215 (2)	
H6B	0.8713 (15)	0.488 (2)	1.0224 (12)	0.032*	
H6A	0.8720 (15)	0.5598 (12)	0.886 (2)	0.032*	
O7	0.83434 (9)	0.21551 (11)	1.10503 (15)	0.0291 (2)	
H7B	0.8305 (17)	0.1320 (10)	1.093 (3)	0.044*	
H7A	0.7743 (10)	0.245 (2)	1.077 (3)	0.044*	
C1	0.48842 (10)	0.38754 (13)	0.82761 (16)	0.0161 (2)	
C2	0.45462 (10)	0.47615 (14)	0.70582 (17)	0.0196 (3)	
H2	0.503151	0.535768	0.662918	0.024*	
C3	0.34987 (10)	0.47766 (14)	0.64666 (17)	0.0198 (3)	
H3	0.326346	0.538419	0.563908	0.024*	
C4	0.27987 (9)	0.38893 (12)	0.71028 (16)	0.0151 (2)	
C5	0.31267 (10)	0.30049 (14)	0.83256 (18)	0.0202 (3)	
H5	0.264213	0.240357	0.874761	0.024*	
C6	0.41732 (10)	0.30097 (14)	0.89257 (17)	0.0199 (3)	
H6	0.440323	0.242335	0.977785	0.024*	
C7	0.60130 (10)	0.38109 (13)	0.88992 (17)	0.0185 (2)	

### Potassium silver 4-carboxybenzenesulfonate salt dihydrate (IV) Atomic displacement parameters (Å^2^)

**Table:**

	*U*^11^	*U*^22^	*U*^33^	*U*^12^	*U*^13^	*U*^23^
K1	0.01622 (10)	0.02482 (11)	0.01888 (11)	−0.00038 (7)	0.00068 (7)	−0.00163 (8)
Ag1	0.01622 (10)	0.02482 (11)	0.01888 (11)	−0.00038 (7)	0.00068 (7)	−0.00163 (8)
S1	0.01276 (13)	0.01278 (13)	0.02192 (17)	−0.00032 (10)	−0.00152 (11)	−0.00075 (11)
O1	0.0136 (4)	0.0304 (5)	0.0319 (6)	−0.0033 (4)	−0.0024 (4)	0.0041 (4)
O2	0.0194 (5)	0.0299 (5)	0.0315 (6)	0.0017 (4)	−0.0049 (4)	0.0061 (5)
O3A	0.0183 (8)	0.0245 (11)	0.0199 (10)	0.0001 (7)	−0.0032 (6)	−0.0016 (8)
O4A	0.0180 (8)	0.0289 (12)	0.0230 (13)	0.0065 (8)	0.0016 (8)	−0.0028 (9)
O5A	0.0213 (8)	0.0176 (7)	0.0393 (17)	−0.0060 (5)	−0.0102 (11)	0.0077 (11)
O6	0.0193 (4)	0.0183 (4)	0.0263 (5)	−0.0034 (4)	−0.0018 (4)	0.0002 (4)
O7	0.0224 (5)	0.0235 (5)	0.0391 (7)	−0.0040 (4)	−0.0120 (5)	0.0060 (5)
C1	0.0142 (5)	0.0155 (5)	0.0186 (6)	0.0009 (4)	0.0003 (4)	−0.0030 (5)
C2	0.0156 (5)	0.0198 (6)	0.0233 (7)	−0.0038 (4)	0.0002 (5)	0.0027 (5)
C3	0.0175 (6)	0.0184 (6)	0.0229 (7)	−0.0017 (5)	−0.0014 (5)	0.0049 (5)
C4	0.0131 (5)	0.0132 (5)	0.0188 (6)	0.0004 (4)	−0.0001 (4)	−0.0021 (4)
C5	0.0154 (6)	0.0198 (6)	0.0253 (7)	−0.0015 (4)	0.0021 (5)	0.0047 (5)
C6	0.0165 (6)	0.0207 (6)	0.0221 (7)	0.0017 (5)	−0.0002 (5)	0.0046 (5)
C7	0.0151 (5)	0.0186 (6)	0.0216 (7)	0.0008 (4)	−0.0005 (5)	−0.0050 (5)

### Potassium silver 4-carboxybenzenesulfonate salt dihydrate (IV) Geometric parameters (Å, º)

**Table:**

K1—O7^i^	2.6233 (12)	O2—C7	1.2167 (17)
K1—O5A^ii^	2.649 (3)	O6—H6B	0.838 (9)
K1—O7	2.7045 (13)	O6—H6A	0.845 (9)
K1—O4A^iii^	2.719 (3)	O7—H7B	0.835 (9)
K1—O6	2.8017 (11)	O7—H7A	0.840 (9)
K1—O5A^iv^	2.968 (3)	C1—C2	1.3903 (19)
K1—O3A^iv^	3.004 (2)	C1—C6	1.3949 (18)
K1—O4A^ii^	3.239 (3)	C1—C7	1.4970 (18)
S1—O3B	1.420 (13)	C2—C3	1.3911 (18)
S1—O5B	1.437 (12)	C2—H2	0.9500
S1—O4A	1.444 (3)	C3—C4	1.3936 (18)
S1—O3A	1.462 (3)	C3—H3	0.9500
S1—O5A	1.473 (3)	C4—C5	1.3891 (19)
S1—O4B	1.523 (12)	C5—C6	1.3918 (19)
S1—C4	1.7759 (12)	C5—H5	0.9500
O1—C7	1.3203 (17)	C6—H6	0.9500
O1—H1	0.79 (2)		
			
O7^i^—K1—O5A^ii^	82.22 (6)	O4B—S1—C4	105.2 (4)
O7^i^—K1—O7	106.02 (4)	C7—O1—H1	109.5
O5A^ii^—K1—O7	171.76 (6)	S1—O3A—K1^v^	95.06 (11)
O7^i^—K1—O4A^iii^	76.03 (7)	S1—O4A—K1^vi^	120.42 (14)
O5A^ii^—K1—O4A^iii^	91.56 (9)	S1—O4A—K1^vii^	87.91 (12)
O7—K1—O4A^iii^	90.63 (6)	K1^vi^—O4A—K1^vii^	131.53 (9)
O7^i^—K1—O6	75.15 (3)	S1—O5A—K1^vii^	112.98 (16)
O5A^ii^—K1—O6	114.66 (7)	S1—O5A—K1^v^	96.27 (12)
O7—K1—O6	68.29 (3)	K1^vii^—O5A—K1^v^	96.81 (9)
O4A^iii^—K1—O6	137.38 (6)	K1—O6—H6B	100.6 (14)
O7^i^—K1—O5A^iv^	169.20 (6)	K1—O6—H6A	127.7 (13)
O5A^ii^—K1—O5A^iv^	96.70 (9)	H6B—O6—H6A	107.1 (19)
O7—K1—O5A^iv^	75.18 (6)	K1^viii^—O7—K1	104.27 (4)
O4A^iii^—K1—O5A^iv^	114.76 (8)	K1^viii^—O7—H7B	107.2 (15)
O6—K1—O5A^iv^	95.76 (6)	K1—O7—H7B	91.6 (16)
O7^i^—K1—O3A^iv^	139.67 (6)	K1^viii^—O7—H7A	131.1 (16)
O5A^ii^—K1—O3A^iv^	71.31 (8)	K1—O7—H7A	109.9 (16)
O7—K1—O3A^iv^	101.65 (6)	H7B—O7—H7A	106 (2)
O4A^iii^—K1—O3A^iv^	74.89 (8)	C2—C1—C6	120.24 (12)
O6—K1—O3A^iv^	143.56 (6)	C2—C1—C7	121.13 (12)
O5A^iv^—K1—O3A^iv^	48.30 (8)	C6—C1—C7	118.63 (12)
O7^i^—K1—O4A^ii^	85.68 (6)	C1—C2—C3	120.12 (12)
O5A^ii^—K1—O4A^ii^	47.18 (8)	C1—C2—H2	119.9
O7—K1—O4A^ii^	131.93 (6)	C3—C2—H2	119.9
O4A^iii^—K1—O4A^ii^	137.14 (3)	C2—C3—C4	119.18 (12)
O6—K1—O4A^ii^	70.37 (5)	C2—C3—H3	120.4
O5A^iv^—K1—O4A^ii^	85.76 (8)	C4—C3—H3	120.4
O3A^iv^—K1—O4A^ii^	97.16 (7)	C5—C4—C3	121.18 (12)
O3B—S1—O5B	110.4 (6)	C5—C4—S1	119.30 (10)
O4A—S1—O3A	112.75 (15)	C3—C4—S1	119.45 (10)
O4A—S1—O5A	111.92 (17)	C4—C5—C6	119.25 (12)
O3A—S1—O5A	112.73 (15)	C4—C5—H5	120.4
O3B—S1—O4B	111.5 (5)	C6—C5—H5	120.4
O5B—S1—O4B	110.2 (6)	C5—C6—C1	120.01 (13)
O3B—S1—C4	109.0 (5)	C5—C6—H6	120.0
O5B—S1—C4	110.3 (4)	C1—C6—H6	120.0
O4A—S1—C4	105.94 (12)	O2—C7—O1	124.52 (12)
O3A—S1—C4	106.77 (11)	O2—C7—C1	122.55 (12)
O5A—S1—C4	106.12 (12)	O1—C7—C1	112.94 (12)

Symmetry codes: (i) *x*, −*y*+1/2, *z*−1/2; (ii) *x*+1, *y*, *z*; (iii) −*x*+1, *y*−1/2, −*z*+3/2; (iv) *x*+1, −*y*+1/2, *z*+1/2; (v) *x*−1, −*y*+1/2, *z*−1/2; (vi) −*x*+1, *y*+1/2, −*z*+3/2; (vii) *x*−1, *y*, *z*; (viii) *x*, −*y*+1/2, *z*+1/2.

### Potassium silver 4-carboxybenzenesulfonate salt dihydrate (IV) Hydrogen-bond geometry (Å, º)

**Table:**

*D*—H···*A*	*D*—H	H···*A*	*D*···*A*	*D*—H···*A*
O1—H1···O6	0.79	1.85	2.6328 (14)	168
O6—H6*A*···O5*A*^vi^	0.85 (1)	2.01 (1)	2.840 (3)	168 (2)
O6—H6*A*···O5*B*^vi^	0.85 (1)	2.01 (2)	2.819 (12)	159 (2)
O6—H6*B*···O4*A*^ix^	0.84 (1)	1.99 (1)	2.824 (3)	176 (2)
O6—H6*B*···O4*B*^ix^	0.84 (1)	1.82 (2)	2.643 (12)	168 (2)
O7—H7*A*···O2	0.84 (1)	1.97 (1)	2.8111 (15)	177 (2)
O7—H7*B*···O3*A*^iii^	0.84 (1)	2.03 (1)	2.838 (3)	162 (2)
O7—H7*B*···O3*B*^iii^	0.84 (1)	1.87 (2)	2.650 (12)	155 (2)

Symmetry codes: (iii) −*x*+1, *y*−1/2, −*z*+3/2; (vi) −*x*+1, *y*+1/2, −*z*+3/2; (ix) −*x*+1, −*y*+1, −*z*+2.
